# Understanding the influence of health systems on women’s experiences of Option B+: A meta-ethnography of qualitative research from sub-Saharan Africa

**DOI:** 10.1080/17441692.2020.1851385

**Published:** 2020-12-07

**Authors:** Shannon M. Williams, Jenny Renjua, Mosa Moshabela, Alison Wringe

**Affiliations:** a Department of Population Health, London School of Hygiene and Tropical Medicine, London, UK; b Department of Epidemiology and Biostatistics, Kilimanjaro Christian Medical College, Moshi, Tanzania; c School of Nursing and Public Health, University of KwaZulu-Natal, Durban, South Africa; d Africa Health Research Institute, Durban, South Africa

**Keywords:** Qualitative research, health systems, pregnant women, sub-Saharan Africa, Option B+

## Abstract

We explored women’s experiences of Option B+ in sub-Saharan African health facility settings through a meta-ethnography of 32 qualitative studies published between 2010 and 2019. First and second-order constructs were identified from the data and authors’ interpretations respectively. Using a health systems lens, third-order constructs explored how the health systems shaped women’s experiences of Option B+ and their subsequent engagement in care. Women’s experiences of Option B+ services were influenced by their interactions with health workers, which were often reported to be inadequate and rushed, reflecting insufficient staffing or training to address pregnant women’s needs. Women’s experiences were also undermined by various manifestations of stigma which persisted in the absence of resources for social or mental health support, and were exacerbated by space constraints in health facilities that infringed on patient confidentiality. Sub-optimal service accessibility, drug stock-outs and inadequate tracing systems also shaped women’s experiences of care. Strengthening health systems by improving health worker capacity to provide respectful and high-quality clinical and support services, improving supply chains and improving the privacy of consultation spaces would improve women’s experiences of Option B+ services, thereby contributing to improved care retention. These lessons should be considered as universal test and treat programmes expand.

## Background

Option B+ is the cornerstone of programmes for the prevention of mother-to-child transmission (PMTCT) of HIV in sub-Saharan Africa, involving life-long initiation of antiretroviral therapy (ART) for pregnant women, regardless of their immunological status. The World Health Organisation (WHO) recommends that PMTCT programmes be integrated within existing antenatal care programmes, with HIV testing and treatment initiation being implemented at the same time as women attend for their routine pregnancy and post-partum care (World Health Organization, 2010b). Option B+ was initially developed and implemented by the Malawian government in 2011, and was subsequently recommended by the World Health Organization (WHO) in 2013 and rolled out by many sub-Saharan African countries thereafter (UNAIDS, 2016; World Health Organization, 2014). In 2018, the implementation of Option B+ enabled 82% [62 – >95%] of pregnant women living with HIV in sub-Saharan Africa to access ART to prevent transmission of HIV to their child (UNAIDS, 2019a). Between 2011 and 2018, these efforts averted an estimated 90,000 new infections amongst exposed infants in East and Southern Africa (UNAIDS, 2019b).

As well as reducing treatment interruptions among women of reproductive age, Option B+ was intended to address health systems challenges that had previously plagued PMTCT programmes by promoting protocol simplification, task-shifting and service decentralisation, thereby enabling higher PMTCT coverage and accelerating progress towards eliminating perinatal infections (Gourlay et al., 2015; Kalua et al., 2017). Option B+ programme data from many sub-Saharan African countries have shown improvements in ART coverage during pregnancy and delivery, resulting in reductions in mother-to-child transmission rates (Kim et al., 2015; UNICEF, 2017). However, these gains may be threatened by poor retention in care, with higher proportions of Option B+ patients becoming lost to follow-up compared to other adults attending ART clinics (Cichowitz et al., 2019; Kieffer et al., 2014), with peaks in attrition occurring immediately after initiation of ART and following delivery of the child (B. Knettel et al., 2018; Tenthani et al., 2016).

Concerns raised about the readiness of some health systems to accommodate increased patient numbers following Option B+ implementation have since been realised (Colvin et al., 2014). In particular, the additional uptake of PMTCT services has not always been matched by corresponding increases in supplies, such as testing kits and appropriate ART regimens (Kieffer et al., 2014; Tenthani et al., 2016). Furthermore, Option B+ implementation accentuated the strain on the workforce in some settings, by resulting in higher patient loads for counsellors and HIV clinic staff, alongside expectations of diversification and role expansion through task-shifting, but without sufficient support and training for such transitions (Tenthani et al., 2016). Evidence is also emerging from some settings of negative impacts of increased PMTCT patient numbers on the quality of counselling, including some instances of coercion and testing without consent (McLean et al., 2017; Wringe et al., 2017). Home-based care workers and other peer or lay health cadres, who have traditionally been involved in promoting linkage to care and conducting tracing visits for patients who are lost to follow up, may also be seeing higher workloads in the absence of additional resources to undertake them (Cataldo et al., 2018). Furthermore, existing challenges in routine monitoring of PMTCT programmes have been exacerbated by more complex data collection requirements for Option B+ that information systems have been unable to cope with, and which can undermine the longitudinal follow-up of patients, including their transition from PMTCT clinics to routine care (Gourlay et al., 2015).

Several systematic reviews of the literature relating to PMTCT have been undertaken, including those investigating retention rates, barriers and facilitators to women’s use of PMTCT services (Colvin et al., 2014; Gourlay et al., 2013; B. Knettel et al., 2018; Yourkavitch et al., 2018) interventions to improve retention in Option B+ (Geldsetzer et al., 2016; B. A. Knettel et al., 2018) and the impacts of PMTCT service delivery on health systems (in the context of Options A and B) (Mutabazi et al., 2017). However, these reviews focused primarily on quantitative data or on limited qualitative findings, and whilst many reviews have shown how implementation of Option B+ has shaped the functioning of the health system, no qualitative reviews have been conducted to explore how the health systems impacts of Option B+ subsequently shape women’s experiences of care. There is now a growing body of individual qualitative studies which have explored the lived experiences of women and their engagement in care. A synthesis of this research will allow a deeper understanding into the mechanisms through which the system shapes women’s experiences. In order to address this gap, we undertook a meta-ethnography of qualitative research on women’s experiences of using Option B+ services in sub-Saharan Africa to understand how they were shaped by health systems conditions, and how this subsequently influenced their engagement with HIV services.This work was undertaken as part of the ‘Strengthening health systems for the application of universal test and treat’ (SHAPE UTT) study. The SHAPE-UTT study is a health systems research project investigating the policy implementation and health systems impacts of HIV test and treat policies in South Africa, Tanzania and Malawi. The overarching aim of the SHAPE-UTT study is to generate evidence on how to strengthen health systems in the context of expanding HIV treatment programmes in sub-Saharan African settings.

## Methods

### Search strategy

This review covers studies that include qualitative data pertaining to women’s experiences of Option B+ services in sub-Saharan Africa published between 1st January 2010 and 31st August 2019, thereby covering the time-period during which Option B+ programmes have been piloted and implemented in the region.

Articles were identified using the Preferred Reporting Items for Systematic Reviews and Meta-Analyses (PRISMA) guidelines for systematic reviews (Moher et al., 2009). Three databases were searched by SW that include a high volume of social science, global health or reproductive health research (PubMed, Popline and Web of Science). Search terms were developed by SW, JR and AW that encompassed all synonyms and spellings related to the following three categories: 1. population (pregnant, post-partum or breastfeeding women); 2. HIV policy (e.g. Option B+, test-and-start, lifelong ART); and 3. geographic area (sub-Saharan Africa) (Table 1). Reference lists from identified systematic and narrative reviews were checked manually by SW to identify relevant publications omitted by the search. Titles and abstracts were imported into EndNote and duplicates were subsequently eliminated. Studies were included in the review if they met the following eligibility criteria: 1. reports primary data; 2. uses qualitative data collection methods; 3. relates to women’s experiences with Option B+; 4. conducted in sub-Saharan Africa; and 5. conducted after January 1st, 2010. Title and the abstract of each paper was reviewed initially by SW to determine their eligibility for full-text review. Following full-text review by SW and JR, we determined if papers met complete eligibility criteria for inclusion in the meta-ethnography.

### Synthesis

We used a meta-ethnographic approach to analyse included studies in chronological order to identify thematic patterns across time (Atkins et al., 2008; Merten et al., 2010; Noblit & Hare, 1988). Stage one of undertaking a meta-ethnography involves identifying first order constructs, which are the perspectives of the study participants. First-order constructs were identified by SW through the direct or indirect quotations study authors presented in results sections of the publications. Stage two involves identifying second-order constructs, which are the themes identified by the researchers of each published study. We identified second-order constructs (SW, JR) through the authors’ analysis of qualitative data collected, as is presented within the results and discussion sections of each publication. In the final stage, the findings were raised to a higher conceptual level through deliberation by the research team (SW, JR, MM, and AW) of overarching themes across all identified studies, whether study findings support or refute each other, changes across the timeline of inquiry and variation across geographic settings. Through this discourse, ‘third order constructs’ emerged that qualify women’s relationships with Option B+ and ways that health system conditions have influenced them.

### Analytical framework

We adopted a health systems lens to explore how women’s experiences of Option B+ were situated within the broader context of the health systems that delivered these services, shaping their engagement in care. Various models have been proposed to conceptualise the elements of a health system, several of which include building blocks or pillars that act together to meet, promote and maintain health in any given community. One of the most commonly used conceptualisations of health systems is the World Health Organization’s Building Block framework which proposes that health systems are composed of Health Service Delivery; Health Workforce; Essential Medicines and Supplies; Health Information Systems; Financing; and Governance (World Health Organization, 2010a). Whilst widely adopted by public health managers and researchers, a recurrent criticism of the framework is that it underplays the dynamic nature of the processes and interactions between the building blocks (Mounier-Jack et al., 2014). Although static frameworks that describe the relationships between the elements of health systems have often been favoured by those aiming to identify interventions to strengthen specific components of the system, some researchers have argued that viewing health systems more broadly as social institutions may help to better understand the nuanced relationship that can both positively and negatively affect care engagement (van Olmen et al., 2012).

We drew on this ‘health systems thinking’ approach in our analysis. A ‘health systems thinking’ approach considers systems as being made up of individuals (patients, health workers and community members), organisations and resources that function together to benefit the overall health of a given community (Savigny & Taghreed, 2010). This approach appreciates the ‘very nature of complex systems as dynamic, constantly changing, governed by history and by feedback, where the role and influence of stakeholders and context is critical, and where new policies and actions (of different stakeholders) often generate counterintuitive and unpredictable effects, sometimes long after policies have been implemented’ (Adam et al., 2012).

### Quality assessment

Two researchers (SW, JR) applied the Brigg’s Institute Checklist for Qualitative Research (Joanna Briggs Institute, 2017) to the eligible publications and scored them individually according to ten criteria which allowed for ‘yes’, ‘no’ or ‘unsure’ responses. Articles were graded by summing up the responses with a score of 1 for each criterion that was met, and a 0 for unsure or unmet criteria. Studies with an agreed score of 5 or greater were included in the analysis. Articles where discrepancies between the scores of the two reviewers, which would have potentially led to ineligibility for inclusion in the review, were passed onto a third researcher (AW) for final review. For articles with score discrepancies where reviewers subsequently reached agreement for inclusion, 0.5 points were awarded for the criterion in question. In the event of articles drawing from the same data, we included the article that achieved the highest quality assessment grade.

## Results

**Figure 1:**
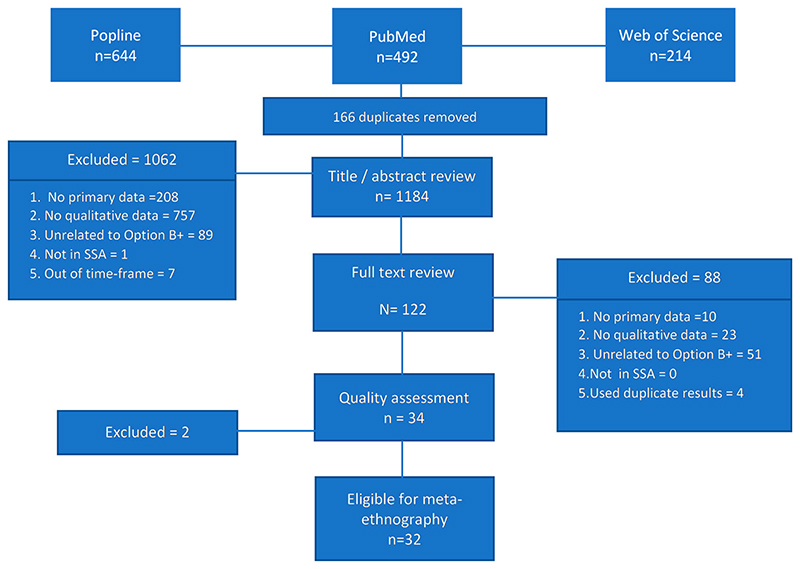
Flow chart showing the identification and selection of the included articles.

Our search identified 1,350 articles, of which 166 were excluded as duplicates. A subsequent 1,062 articles were excluded during the title or abstract review as not meeting eligibility criteria for inclusion (Figure 1). One hundred and twenty-two studies were selected for full text review, of which 84 were excluded for not meeting eligibility criteria. We further excluded another four studies that used results duplicated in another paper. In total, 34 studies were included in our quality assessment.

### Quality assessment

The average quality score of all included studies was 7.56 / 10 points. Ninety-four percent of studies scored five points or higher with two studies not meeting the quality threshold (scores = 4 and 2). The remaining 32 papers were included in our analysis (Appendix A). The Brigg’s criterion included most consistently in publications was a statement on ethical approval (criterion 9, included in 100%). Criteria that were most frequently unmet were inclusion of a statement regarding the researchers’ background or perspective (criterion 6, absent in 66% of studies) and reflection on the researcher’s relationship with, or influence on, study participants (criterion 7, absent in 82% of studies).

### Characteristics of included studies

**Figure 2:**
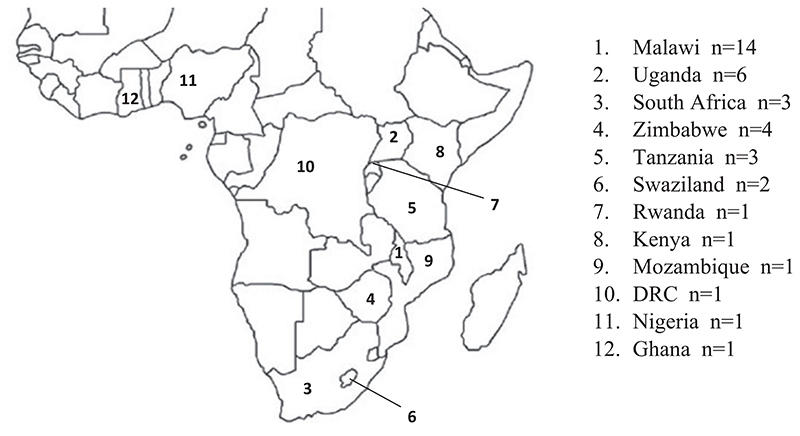
Geographic location of the studies included in the meta-ethnography.

Of the 32 included studies, two were published in 2014, six in 2016, fifteen in 2017, five in 2018, and four in 2019. Studies covered a nine-year period during which Option B+ was rolled out across sub-Saharan Africa (Appendix A). Research was conducted in 38 settings in 12 different countries. The country where most published studies were undertaken was Malawi (n=14), followed by Uganda (n=6), Zimbabwe (n=4), South Africa (n=3), Tanzania (n=3), Swaziland (n=2), Rwanda (n=1), Kenya (n=1), Mozambique (n=1), the Democratic Republic of Congo (n=1), Nigeria (n=1) and Ghana (n=1) (Figure 2).

### Themes

Thirty first-order constructs detailing women’s experiences of Option B+ care were identified in the included studies, with minimal variation across the timeline of enquiry. Sixteen second-order constructs were identified (Table 2), describing how women’s experiences of Option B+ have been shaped by the health system (second-order constructs are underlined and first-order constructs are presented as quotations in italics in the subsequent sections of the results).

**Figure 3:**
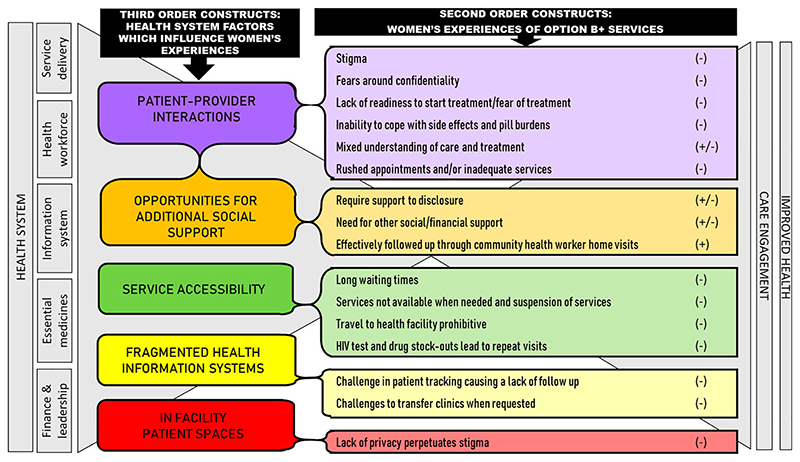
Conceptual framework illustrating how women’s experiences (2nd order constructs) are shaped by health system elements (3rd order constructs) subsequently influencing care engagement and outcomes.

In the following sections we describe the five third-order constructs that emerged from the data to explain how health systems processes and dynamics shape women’s experiences of care in ways which are not always intended by policy. The five third order constructs include: patient-provider interactions, opportunities for additional social support, service accessibility, fragmented health information systems and in-facility patient spaces (Figure 3).

### Patient-provider interactions

Relationships between the patient and the provider appeared to have the strongest influence on women’s reported experiences of, and engagement in, care. Poor interactions with health providers perpetuated *stigma* and created *fears around confidentiality* that failed to support women’s readiness to start ART (Buregyeya et al., 2017; Cataldo et al., 2017; Doherty et al., 2017; Elwell, 2016; Flax et al., 2017a; Flax et al., 2017b; Gill et al., 2017; Helova et al., 2017a; Mamba & Hlongwana, 2018; Masereka et al., 2019; McMahon et al., 2017; Mulamba et al., 2017; Napúa et al., 2016; Nkhata et al., 2016; Phiri et al., 2018).

‘Sometimes the health workers that test you or counsel you then judge us for our actions or even shout at us. For example, they will sometimes say, how I could be negligent about my life and expose myself to the virus? … It‘s not the words per se, but the tone in which they talk to you … if they could talk to us in a way that is comforting.’ (Pregnant Woman with HIV, Swaziland) (Mamba & Hlongwana, 2018)

Studies reported how inadequate staffing, in terms of overall numbers as well as in the training they had received, lead to *rushed appointments and/ or inadequate services* (Chadambuka et al., 2018; Doherty et al., 2017; Hanrahan & Williams, 2017; Helova et al., 2017; Katirayi, et al., 2016a; Kweyamba et al., 2018; Laar et al., 2018; McMahon, S., 2017; Mulamba et al., 2017; Napúa et al., 2016; Nkhata et al., 2016; Phiri et al., 2018).

‘It’s the service providers’ problem! They didn’t tell me anything. They should have told me to go to some place for services. I would have followed their instructions!’ (Mother with HIV, Tanzania) (McMahon et al., 2017)

As well as directly impacting on patients’ experiences, these insufficiencies lead staff to feel overwhelmed, burned out, or frustrated in the face of being expected to deliver care without the necessary resources to do so (Chadambuka, et al., 2018; Doherty, et al., 2017; Hanrahan & Williams, 2017; Helova, et al., 2017; Katirayi, et al., 2016a; Kweyamba, et al., 2018; Laar, et al., 2018; Mulamba, et al., 2017; Napúa, et al., 2016; Nkhata, et al., 2016; Phiri, et al., 2018).

‘The person attending to this client is having burn out, so that information is not given correctly. Like giving medication without following how the client will use the medication.’ (HIV Testing Counsellor, Kenya) (Helova et al., 2017)

In some settings, care delivery was supported by community health workers or peer mentors to reduce the burden of work on qualified nurses and midwives (Cataldo, et al., 2018; Doherty et al., 2017; Gugsa et al., 2017; Masereka et al., 2019; Phiri et al., 2018). However, these lay cadre were not always adequately trained to assist with clinical tasks, such as testing or ART counselling, thus were not capable of filling workforce gaps that require more training and certification (Cataldo et al., 2018; Doherty et al., 2017; Gugsa et al., 2017; Hanrahan & Williams, 2017; Napúa et al., 2016; Nkhata et al., 2016; Phiri et al., 2018).

‘I know ART has come a long way, and it can be provided by a whole host of healthcare workers, but one still needs decent clinical care expertise (International Implementing Partner, Uganda) (Doherty et al., 2017)

Provider perspectives and the way in which they relayed health messages lead to *mixed understandings of care and treatment* that affected care engagement (Buregyeya et al., 2017; Chadambuka et al.,2018; Doherty et al., 2017; Elwell, 2016; Erekaha et al., 2018; Flax et al., 2017; Helova et al., 2017; Katirayi, et al., 2016a; Masereka et al., 2019; McMahon et al., 2017; Phiri et al., 2018). Many studies reported how health workers emphasised the need for women to remain ART adherent in order to protect their unborn baby (Black et al., 2014; Buregyeya et al., 2017; Cataldo et al., 2018; Chadambuka et al., 2018; Clouse et al., 2014; Doherty et al., 2017; Flax et al., 2017a; Flax et al., 2017b; Gill et al., 2017; Gugsa et al., 2017; Helova et al., 2017; Katirayi, et al., 2016a; McLean et al., 2017; Mulamba et al., 2017; Nkhata et al., 2016; Phiri et al., 2018; Rosenberg et al., 2017; Sariah et al., 2019; Zhou, 2016). However, limited post-partum psychosocial counselling in many settings often led to faltering ART adherence, and loss-to-follow up (LTFU) beyond delivery.

‘I tested my baby, and she was HIV-negative, then I thought I was fit and able to carry on with my daily activities, so I decided to stop taking the medication.’ (Mother with HIV, Tanzania) (Sariah et al., 2019)

Other studies highlighted that the quality of counselling was particularly problematic in terms of supporting clients’ *ability to cope with side effects and pill burden* or HIV treatment fatigue (Buregyeya et al., 2017; Chadambuka et al., 2018; Erekaha et al., 2018; Flax et al., 2017b; Gill et al., 2017; Gugsa et al., 2017; Laar et al., 2018; Katirayi, L., 2016b; Katirayi, et al., 2017; Mamba & Hlongwana, 2018; McMahon et al., 2017; Nkhata et al., 2016; Phiri et al., 2018; Sariah et al., 2019; Zhou, 2016).

‘I came and explained about the side effects of the drug that I felt. They responded that if it is the first time, you are likely to experience those side effects, but I was not feeling better for the whole month, and if I decide to miss the treatment, was feeling better. Then later on I decided to quit the treatment; I didn’t take the drugs.’ (Mother with HIV, Malawi) (Zhou, 2016)

The way in which health workers conveyed counselling messages affected women’s experiences of care, including their ability to cope with stress, depression, low self-worth and experiences of stigmatisation (Erekaha et al., 2018; Flax et al., 2017a; Gill et al., 2017; Gugsa et al., 2017; Mamba & Hlongwana, 2018; McMahon et al., 2017; Sariah et al., 2019). Providers who presented HIV as a chronic, manageable condition often left women feeling more positive about their experiences with HIV services and more likely to adhere to ART (Black et al., 2014; Buregyeya et al., 2017; Chadambuka et al., 2018; Doherty et al., 2017; Elwell, 2016; Gill et al., 2017; Gugsa et al., 2017; Katirayi, et al., 2016a; Katirayi, et al., 2016b; Phiri et al., 2018; Rosenberg et al., 2017).

‘The nurses helped me. The nurse who was working here, we knew each other and were friends. And she helped me and convinced me that anyone may have this problem and it is good to take the medicine … in general there is no problem; the important thing is to take the medicine.’ (Mother with HIV, Rwanda) (Gill et al., 2017)

Women who tested HIV positive at their first ANC visit faced a triple burden which included balancing the responsibility for attending ANC, coping with an HIV diagnosis and becoming ready to initiate ART. Women’s *lack of readiness to start treatment or fear of treatment* often required ongoing psychosocial support, which was overlooked or not provided due to provider time constraints (Black et al., 2014; Cataldo et al., 2017; Flax et al., 2017a; Gill et al., 2017; Gugsa et al., 2017; Helova et al., 2017; Katirayi, et al., 2016a; Katirayi, L., 2016b; Kweyamba et al., 2018; Laar et al., 2018; Mamba & Hlongwana, 2018; McLean et al., 2017; McMahon et al., 2017).

‘The first counselling is not enough because it is not easy for someone to have come to ANC, have her blood tested, be found to be HIV positive and at the same time be told to initiate to ART. As a result, more women just accept it to please us and [so we will] let them go, but when they go home they don’t take the medicine or disclose to their husbands.’ (Healthcare Worker, Uganda) Katirayi, et al., 2016b)

### Opportunities for additional social support

Women across many studies reported *needs for social or financial support* surrounding ongoing care engagement that was unavailable to them (Bengston, et al., 2020; Black, et al., 2014; Buregyeya, et al., 2017; Cataldo, et al., 2017; Cataldo, et al., 2018; Clouse, et al., 2014; Elwell, 2016; Flax, et al., 2017a; Flax, et al., 2017b; Gill, et al., 2017; Gugsa, et al., 2017; Helova, et al., 2017; Katirayi, et al, 2016a; Katirayi, et al., 2016b; [Bibr R29][Bibr R38]; Masereka et al., 2019; McLean, et al., 2017; McMahon, et al., 2017; Nkhata, 2016; Sariah, 2019).

‘As the female I currently don‘t have a job and I‘m depending upon my partner financially … if there was a way that the clinic offered some form of financial support after testing HIV positive, I would be able to support myself even after he had left me.’ (Pregnant Woman with HIV, Swaziland) (Mamba & Hlongwana, 2018)

In particular, many studies discussed the importance of partner support and how women *require support to disclose* to their partners. Some health facilities sent letters inviting partners to attend ANC appointments or to encourage partner testing; others tried to increase their to appeal to partners by offering men’s areas in waiting rooms (Chadambuka et al., 2018; Flax et al., 2017b; Helova et al., 2017; Kweyamba et al., 2018; Phiri et al., 2018; Rosenberg et al., 2017).

‘Before they told us the results with my husband, they asked us a few questions. They asked him if I tested positive could he accept … can he continue being with me or not? He accepted that he would stay with me. They asked me as well. I said I would be with him still, so he accepted, and we are still together’ – (Mother with HIV, Malawi) (Phiri et al., 2018)

However, both health workers and women reported that Option B+ counselling services were frequently inadequate in terms of addressing conflict that could arise between couples related to disclosure, such as abuse, neglect or divorce (Bengtson et al., 2020; Black et al., 2014; Buregyeya et al., 2017; Cataldo et al., 2017, 2018; Clouse et al., 2014; Elwell, 2016; Flax et al., 2017a; Flax et al., 2017; Gill et al., 2017; Gugsa et al., 2017; Helova et al., 2017; Katirayi, L. 2016a; Katirayi, L., 2016b; Mamba & Hlongwana, 2018; Masereka et al., 2019; McLean et al., 2017; McMahon et al., 2017; Nkhata et al., 2016; Sariah et al., 2019).

In addition to the care provided in health facilities, multiple studies identified the need for support for pregnant women living with HIV to be *effectively followed-up through community health worker home visits* (Cataldo et al., 2018; Doherty et al., 2017; Kweyamba et al., 2018; Masereka et al., 2019; Phiri et al., 2018). In some settings, community health workers provided psychosocial and practical support, including sending appointment reminders by text message, visiting women in their home or acting as ART adherence role models.

### Services accessibility

Health workers recognised that service access was affected by long waiting times with this being attributed to limited availability of qualified staff (Buregyeya, et al., 2017; Doherty, et al., 2017; Hanrahan, B., 2017; Helova, et al., 2017; Kweyamba, et al., 2018; Laar, et al., 2018; Mulamba, et al., 2017; Napúa, et al., 2016; Nkhata, et al., 2016).

‘The staffs we are getting are newly recruited who have just completed school without counseling experience. There is too much workload on the few experienced ones around thus resulting in long waiting time for our clients.’ (Healthcare Worker, Ghana) (Laar et al., 2018)

Women reported how wait times led to feeling conflicted in terms of competing responsibilities, such as childcare or work commitments (;Cataldo, et al., 2017; Flax et al., 2017a; Flax et al., 2017b; Helova et al., 2017; Laar et al., 2018; McMahon et al., 2017; Napúa et al., 2016; Nkhata et al., 2016; Phiri et al., 2018). Some studies reported that women felt *services were not available when they needed them*.

‘The past two months I have not been taking the medicine, because the day I went there, it was the wrong day and I am mostly busy selling vegetables.’ (Mother with HIV, Malawi) (Zhou, 2016)

Women, particularly in more rural areas, reported that *travel to health facilities* and the subsequent corresponding time and financial costs incurred impacted on their access to reliable and ongoing services. (Cataldo et al., 2018; Doherty et al., 2017; Flax et al., 2017a; Gugsa et al., 2017; Laar et al., 2018; Phiri et al., 2018; Rosenberg et al., 2017; Sariah et al., 2019).

‘Sometimes I want to come to the hospital, but due to lack of transport money I don’t. If money is available it may be easy for me to take the drugs […] there are no relatives around [to provide transportation assistance], as they are all in the village and I stay with my husband. So, if he finds money, I can come to collect the drugs.’ (Pregnant Woman with HIV, Malawi) (Gugsa et al., 2017)

In some studies, a reported lack of funding impacted on women’s access to care through limited opening hours and the *suspension of services,* particularly supplementary psychosocial or peer support services (Doherty et al., 2017; Kweyamba, et al., 2018; Mulamba et al., 2017; Napúa et al., 2016; Zhou, 2016).

‘Yeah we have them [mentor mothers] […] What their input has been doing in this community has been really great. And their failure to renew means we are just going back to where we were in the past.’ (Healthcare Worker, Uganda) (Doherty et al., 2017)

Access to services was further curtailed through persistent *HIV test and drug stock-outs* (i.e. ART and other essential medications) which resulted in the need for women to attend multiple clinic visits (Doherty et al., 2017; Elwell, 2016; Flax et al., 2017b; Hanrahan & Williams, 2017; Helova et al., 2017; Laar et al., 2018; McLean et al., 2017; Nkhata et al., 2016; Phiri et al., 2018).

‘With Option B+, there is a new guideline with new regimen for drugs. Then the challenge we have is that when we make phone calls to the higher authority. they tell you that the drug is not available. In fact, they tell you not to initiate many people on it because the drug is still not available …’ (Nurse, Kenya) (Helova et al., 2017)

### Fragmented health information systems

The mechanisms adopted and implemented to trace and contact women who had previously disengaged presented *challenges in patient tracking causing lack of follow-up* from care (Bengtson et al., 2020; Buregyeya et al., 2017; Cataldo et al., 2017, 2018; Doherty et al., 2017; Gugsa et al., 2017; Hanrahan & Williams, 2017; Helova et al., 2017; Laar et al., 2018; Napúa et al., 2016; Rosenberg et al., 2017). Barriers to patient tracing included sub-optimal record-keeping, perpetuated by the use of paper health records, which required storage space. The lack of space reported in many facilities led to challenges in locating records.

Women described *challenges transferring clinics* related to missing or inadequate paperwork, including health passports, which contributed to their inability to receive services and to subsequently disengaging from care (Bengtson et al., 2020; Clouse et al., 2014; Flax et al., 2017a; Flax et al., 2017b; Gugsa et al., 2017; Hanrahan & Williams, 2017; Helova et al., 2017; McLean et al., 2017; McMahon et al., 2017; Mulamba et al., 2017; Nkhata et al., 2016; Sariah et al., 2019).

‘First, they told me they won’t give me any drug without the transfer letter. I asked them if I should miss my drugs because of the transfer letter. They wondered how they could assist me because you must have the letter with you. I wondered how I could walk everywhere with the whole file.’ (Mother with HIV, Kenya) (Helova et al., 2017)

Some studies reported that the process of upgrading to electronic health records exacerbated health workforce challenges because of the additional training and data entry requirements (Bengtson et al., 2020; Buregyeya et al., 2017; Cataldo et al., 2017, 2018; Doherty et al., 2017; Gugsa et al., 2017; Hanrahan & Williams, 2017; Helova et al., 2017; Laar et al., 2018; Napúa et al., 2016; Rosenberg et al., 2017).

### In facility patient spaces

Within the clinic setting, the limited size of consultation rooms and waiting areas made it challenging to accommodate and ensure suitable spaces for providing care to HIV patients. Women were concerned about other patients overhearing what was said in their consultations, being seen in HIV patient areas by patients or health workers they knew personally or being called out by name in waiting areas to pick up their medications.

‘ … the physical facilities, the staffing establishment was never designed to see the same patient coming in every day for the rest of their life. It was never designed to have progressively increasing numbers of individuals coming in for that service … and then you will see those effects in the quality of overall care that has been provided.’ (International Implementing Partner, Uganda) (Doherty et al., 2017)

This lack of space negatively influenced women’s experiences, with patients and providers reporting that the *lack of privacy* perpetuated anticipated and enacted stigma (Doherty et al., 2017; Flax et al., 2017; Helova et al., 2017; Laar et al., 2018; Masereka et al., 2019; Mulamba et al., 2017; Phiri et al., 2018).

‘For example, let’s say I am a client and I see my neighbor in the queue for collecting ARVs; I will not join the queue but I will just be walking about the building …’(Mentor Mother) (Bengtson et al., 2020)

The lack of integrated services for HIV were also seen to contribute to different manifestations of stigma, with women reportedly disliking attending appointments in areas specifically designated for HIV services (Bengtson et al., 2020; Buregyeya et al., 2017; Cataldo et al., 2018; Elwell, 2016; Flax et al., 2017a; Flax et al., 2017b; Gill et al., 2017; Helova et al., 2017;Kweyamba, M., 2018 ; Masereka et al., 2019; McLean et al., 2017; McMahon et al., 2017; Sariah et al., 2019).

## Discussion

We performed a meta-ethnography of qualitative research that explored women’s experiences with Option B+ in sub-Saharan African settings to understand how their engagement in care was influenced by the health system within which services were delivered. Thirty-two studies were included from 30 settings in 12 countries covering a nine-year time-span. We found that the experiences of women across multiple settings were comparable, suggesting similar health service delivery mechanisms and subsequent constraints within these resource limited health systems across different countries. We also found little to suggest that health systems had sufficiently evolved to address these challenges to improve women’s experiences of Option B+ over the study period. Using a health systems thinking approach, our meta-ethnography highlighted various elements of the system that shape care experiences. These issues related to the interactions women had with the health workers, the opportunities for social support, service accessibility, systems to trace within and link between facilities and the patient spaces within the facilities.

Option B+ has been recognised as a ‘paradigm shifting innovation’ which has re-shaped HIV care and treatment for pregnant women across many sub-Saharan African countries (Kalua et al., 2017). However, the increased volume of women initiating ART and needing to be retained in care has undoubtedly placed pressures on health systems. Our findings support recent studies which report that whilst Option B+ has simplified PMTCT services for both patients and providers, implementation continues to be undermined by health systems inadequacies which negatively impact women’s experiences and engagement in care (DiCarlo et al., 2019; Phiri et al., 2019).

The investments made in scaling up Option B+ and antiretroviral therapy to women living with HIV in a bid to eliminate mother-to-child transmission will not realise their potential if women’s negative experiences lead to them dropping out of care. The focus on providing increasing quantities of drugs needs to be adequately accompanied by better efforts, and greater investments in health system strengthening. This should involve increasing the capacity of the workforce to enable providers to offer quality services and address reporting challenges to better capture and track patient data, and improving the in-facility patient spaces to ensure privacy. These findings are particularly relevant as countries expand test and treat approaches beyond PMTCT to include all individuals living with HIV.

In many countries, efforts have been made to compensate for inadequate numbers of health workers for a growing patient population by adopting task shifting and sharing (Callaghan et al., 2010; Woldie et al., 2018). While these efforts reduce provider workloads and subsequently increase service availability and uptake (Flick et al., 2019), they can also result in health workers being assigned work they are not qualified or supported to do (Munga et al., 2012), potentially undermining the confidence and subsequent trust that women have in their health provider. Our findings support the need for training of health workers to move beyond an understanding of guidelines and technical skills, to include education and mentoring that helps them to build positive, empathetic relationships based on trust with their patients.

Our findings show how the elements of the health system shape women’s experiences in multiple overlapping ways and support the need to take a holistic approach to improve the resilience of the heath system as a whole (Savigny & Taghreed, 2010). Increased client numbers require strengthened psychosocial support structures within the health facility and the community, which need to be robust and responsive to the heterogeneity of this larger population of pregnant women. In order to ensure positive experiences for women and the population more broadly, health systems need to be dynamic and deliver services that recognise how care engagement is situated within the broader context of a person’s life (Renju et al., 2017). Studies applying theories of practice to HIV care strongly endorse the need for the delivery of patient centred, differentiated care to ensure that the needs of individuals are met and subsequent care engagement is promoted (Skovdal et al., 2017). Our findings join this call and further promote the need to change the way we frame our health systems. We need to move away from a focus on ‘building blocks’ as static elements within a system, (Mounier-Jack et al., 2014; World Health Organization, 2010a) to consider the fluid nature of women’s care engagement and the interlinked and overlapping ways that elements of the system can influence her experiences.

Our study provides a timely compendium of studies and subsequent lessons drawn from nearly 10 years of Option B+ implementation across sub-Saharan Africa. These lessons are likely to be applicable as universal test and treat for the general population are widely implemented across the same region. However, our meta-ethnography has various limitations inherent to the meta-ethnographic approach that should be considered when interpreting our findings. Firstly, our search was limited to studies published in English and could potentially bias findings towards countries where English is more widely used (specifically East and Southern Africa). Secondly, our quality assessment found an almost uniform absence of detail on the relationship between the researchers and study subjects (reflexivity). The omission of this relationship in reports hampers a clear understanding of the context in which the research was conducted, the perspective taken and the methods adopted to take the presence of the researcher into account when presenting the findings. In our review, the lack of reflexivity in the included studies meant that it was difficult to assess the extent to which the discussions around clinic attendance were shaped by the nature of the relationships between the field workers and the participants. Similar concerns have been found in other reviews of qualitative research from African settings (Lytvyn et al., 2017). However, despite the absence of statements on reflexivity in many studies, we noted that authors from sub-Saharan Africa were well represented in the identified papers. We further noted that the studies included in our meta-ethnography were largely focused on the barriers, deterrents and challenges women face surrounding care engagement and ART adherence. Although some papers did explore motivating factors or facilitators, these same papers also identified challenges; while the papers identifying challenges did not always present factors that have assisted with women’s care retention. It is likely that this omission is a reporting bias in which authors do not focus on discussing when the system is functioning as it should be, but tend to focus more strongly on when the system is functioning sub-optimally. Finally, our study would be limited by any inherent limitations present in the qualitative studies included, for example the effects of any social desirability bias.

In conclusion, our meta-ethnography of women’s experiences of Option B+ highlights the pressures that a public health and ‘one-size fits all’ policy places on the health system and conversely how these health system pressures shape women’s experiences including care engagement, retention and outcomes. As universal test and treat programmes are implemented across the continent, increased efforts are needed to balance the pressure of more people in care with stronger, more resilient and dynamic health systems. Such efforts will enable high coverage and positive care experiences, to in turn improve care engagement and subsequent population level impact.

## Supplementary Material

Supplementary Material

## Figures and Tables

**Table 1:** Search Strategy

Search category	Terms
Population	pregnant OR natal OR "post-partum" OR partum OR antenatal OR breastfeed* OR PMTCT OR "prevention of mother to child transmission" OR "mother-to-child" OR mother OR maternal OR lactating OR postnatal OR "post-natal"
HIV Policy	option b+ OR "option b plus" OR "test and start" OR "test-and-start" OR "test and treat" OR "test-and-treat" OR "lifelong treatment" OR "lifelong ART" OR "lifelong antiretroviral treatment" OR "life-long ART" OR "life-long antiretroviral treatment" OR "rapid ART" OR "rapid antiretroviral treatment" OR "rapid initiation" OR "same day treatment" OR "treatment as prevention" OR "treatment-as-prevention" OR "same-day-treatment" OR "universal ART" OR "universal antiretroviral treatment"
Geographic Area	"sub-Saharan Africa" OR Angola OR Benin OR Botswana OR Burkina Fas OR Burundi OR Cameroon OR Cape Verde OR Central African Republic OR Chad OR Comoros OR Congo OR Democratic Republic of Congo OR Cote d’Ivoire OR Djibouti, Equatorial Guinea, Eritrea OR Ethiopia OR Gabon OR The Gambia OR Ghana OR Guinea OR Guinea-Bissau OR Kenya OR Lesotho OR Liberia OR Madagascar OR Malawi OR Mali OR Mauritania OR Mauritius OR Mozambique OR Namibia OR Niger OR Nigeria OR Reunion OR Rwanda OR Sao Tome and Principe OR Senegal OR Seychelles OR Sierra Leone OR Somalia OR South Africa OR Sudan OR Swaziland OR Tanzania OR Togo OR Uganda OR Western Sahara OR Zambia OR Zimbabwe

**Table 2:** Mapping second order constructs to a summary of first order constructs and illustrating in which publications they appeared.

Year of research timeline	2011		2012			2013				2014								2015									2016		2017 - 2018				2nd order constructs
**First order constructs**	1	2	3	4	5	6	7	8	9	10	11	12	13	14	15	16	17	18	19	20	21	22	23	24	25	26	27	28	29	30	31	32	
**1** Health worker attitudes surrounding HIV as a barrier or facilitator			**X**		**X**	**X**					**X**		**X**				**X**		**X**	**X**		**X**	**X**		**X**		**X**		**X**		**X**	**X**	**Stigma**
**2** Concerns over lack of confidentiality			**X**		**X**			**X**			**X**		**X**						**X**	**X**							**X**			**X**			**Confidentiatliy**
**3** Feeling rushed into treatment after diagnosis; patients wanting more time	**X**			**X**	**X**		**X**	**X**					**X**		**X**				**X**	**X**	**X**				**X**			**X**				**X**	**Lack of readiness to start treatment or fear of treatment**
**4** Fear of not being able to take medication for life, or worried the pills will stop working				**X**						**X**							**X**			**X**		**X**			**X**						**X**	**X**	
**5** Worried about the side effects prevents starting ART		**X**		**X**	**X**									**X**						**X**		**X**			**X**		**X**				**X**	**X**	**Inability to cope with side e**ff**ects or**
**6** Struggled with side effects, may or may not have received counselling to manage				**X**	**X**					**X**	**X**			**X**			**X**	**X**		**X**		**X**			**X**			**X**			**X**		**pill burden**
**7** Don’t like taking the medication (size, number, taste)											**X**			**X**			**X**			**X**	**X**											**X**	
**8** Feeling insufficiently informed by the provider, not getting enough information			**X**	**X**	**X**		**X**				**X**				**X**		**X**	**X**	**X**			**X**		**X**						**X**			**Mixed understanding of care and treatment**
**9** Community outreach (targeted towards men and pregnant women) reinforced testing, adherence and HIV education				**X**								**X**		**X**		**X**		**X**					**X**	**X**	**X**								
**10** Fear oftransmission to baby or partner; LTFU after baby tests negative	**X**	**X**				**X**	**X**			**X**	**X**		**X**		**X**	**X**	**X**	**X**	**X**	**X**		**X**	**X**	**X**	**X**	**X**	**X**				**X**		
**11** Use of role models to present diagnosis as a manageable condition rather than death sentence	**X**		**X**	**X**			**X**				**X**					**X**		**X**		**X**		**X**		**X**	**X**								
**12** Received different messages by different providers; or had a different experience last pregnancy *(want short-term regimine)*				**X**	**X**		**X**			**X**					**X**									**X**									
**13** CHW (volunteers) can alleviate system burden, but may be inadequately trained		**X**							**X**			**X**										**X**	**X**	**X**	**X**								**Rushed appointments and/or inadequate services**
**14** Health workers over-burdened resulting in limited counselling, poor attitudes or service delays		**X**		**X**					**X**			**X**	**X**					**X**			**X**	**X**		**X**		**X**		**X**					
**15** Clinic facilitated couple engagement or supported partner disclosure													**X**	**X**		**X**		**X**			**X**	**X**					**X**						**Require support with disclosure**
**16** Fear of confiding diagnosis because of stigma, neglect, loss of social or economic support - unsupported by any clinic initiatives	**X**	**X**	**X**	**X**	**X**	**X**	**X**	**X**			**X**		**X**	**X**	**X**				**X**	**X**			**X**		**X**		**X**		**X**	**X**	**X**	**X**	**Need for other social or** fi**nancial support**
**17** Group counselling or ongoing counselling offered by clinic or community health services																	**X**				**X**	**X**		**X**				**X**		**X**		**X**	
**18** Mental health needs, including depression / despair, unaddressed					**X**												**X**		**X**	**X**					**X**						**X**	**X**	
**19** CHW or volunteers offer home visits or act as peer mentors																					**X**	**X**	**X**	**X**						**X**			**E**ff**ectively followed-up through community health workers**
**20** Frequency of appointments needed and long waiting times makes attendance too time consuming		**X**			**X**			**X**	**X**				**X**						**X**			**X**					**X**	**X**					**Services not available when needed**
**21** Cancelled services due to unreliable funding; interventions stopped, clinic hours reduced, inadequate supplies									**X**	**X**											**X**			**X**		**X**							
**22** Travel to and from health facilities takes too long or costs too much																**X**			**X**			**X**	**X**	**X**	**X**			**X**			**X**		**Travel to health facility prohibitive**
**23** Challenges in access to quality care from trained workers because of long waiting times		**X**							**X**		**X**	**X**	**X**								**X**			**X**		**X**		**X**					**Long waiting times**
**24** Multiple visits needed for HIV test, or medicines, as out of stock or without the laboratory capacity for testing		**X**	**X**									**X**	**X**		**X**							**X**		**X**			**X**	**X**					**Need for repeat visits**
**25** Challenges transfering and receiving care from different clinics or with health papers / passports		**X**			**X**	**X**						**X**	**X**		**X**				**X**		**X**				**X**	**X**	**X**		**X**		**X**		**Challenges transfering clinics when requested**
**26** Patient tracking: system challenges, length of time it takes to input data and upgrade system								**X**	**X**			**X**	**X**			**X**							**X**	**X**	**X**			**X**	**X**				**Challenge in patient tracking**
**27** Crowded waiting rooms, long wait times, unwanted disclosure as a result of no privacy.													**X**						**X**			**X**		**X**		**X**		**X**		**X**			**Lack of privacy**
**28** Fear of unwanted disclosure due to HIV ward/ HIV care facilities clearly marked / too obvious			**X**		**X**						**X**		**X**		**X**				**X**	**X**	**X**		**X**				**X**		**X**	**X**	**X**		

## Data Availability

Data will be available for the next five years upon request to the study PI: Alison.Wringe@lshtm.ac.uk
