# Supplementary material for: Understanding the influence of health systems on women’s experiences of Option B+: A meta-ethnography of qualitative research from sub-Saharan Africa
Source: Glob Public Health. Author manuscript; Available in PMC 2022 Jul 2. (PMC7612946; doi:10.1080/17441692.2020.1851385)
Supplement: Supplementary Material [file EMS146173-supplement-Supplementary_Material.docx]

# Appendix A. Study Characteristics

| Study # | Year of research | Author | Research location | Setting | Method | Population | Aim | QA score |
| --- | --- | --- | --- | --- | --- | --- | --- | --- |
| 1 | 2011 | (Black et al., 2014) | Cape Town, South Africa | Midwife-driven ANC clinic | SSI | Rapid ART patients and service providers. | To understand the acceptability of rapid initiation of lifelong ART by pregnant women. | 8 |
| 2 | 2011-2012 | (Nkhata et al., 2016) | Malawi and Zimbabwe | Rural, semi-urban and urban sites at the primary, secondary and tertiary health facility level | SSI, FGD | HCWs who provide ART services. | To compare staffing levels, turnover and perceptions and experiences of staff involved in the delivery of decentralized ART services in the three countries. | 7 |
| 3 | 2012 | (Elwell, 2016) | Southern Malawi | PMTCT program at an urban hospital and 3 health centres (1 urban and 2 rural) | SSI, FGD | Current and LTFU PMTCT patients, HCW, community leaders | To examine how relations within the household, community, and clinic affect Malawian women’s participation in PMTCT programs. | 6.5 |
| 4 | 2012 | (Leila Katirayi et al., 2016) | Four regions in Swaziland: | Sites with the highest number of annual deliveries in both urban and rural locations | Short-answer questionnaires, FGD | Pregnant and breastfeeding women with HIV, nurses | to understand the challenges related to ART initiation among pregnant women beginning a lifelong commitment to treatment | 7.5 |
| 5 | 2011 - 2013 | (McMahon et al., 2017) | Morogoro region, Tanzania | 2 urban and 2 rural Care and Treatment Centres (CTCs) | IDI | LTFU postpartum women with HIV | To understand why women who start PMTCT fail to complete it by relying primarily or exclusively on data from women who have disengaged from care and are not enrolled in a trial. | 10 |
| 6 | 2013 | (Clouse et al., 2014) | Johannesburg, South Africa | Urban ANC clinic | SSI, FGD | Pregnant & postpartum women living with HIV | To identify challenges to retention in care during the postpartum period faced by HIV-positive women initiating ART | 7.5 |
| 7 | 2013 | (L Katirayi et al., 2016) | Malawi | 1 urban, 1 rural &  2 peri-urban hospitals | FGD,IDI | Pregnant / lactating women with HIV & HCWs | To explore the critical issues shaping acceptance to ART initiation and adherence among women under Option B+. | 7.5 |
| 8 | 2013 | (Cataldo et al., 2017) | Malawi | 6 health facilities in SE, SW and central zones | IDI, FGD | Patients enrolling in Option B+; HCW | To elicit local perceptions of Option B+ among HCWs and women who have recently enrolled in the program and to document the nature of the support provided. | 7 |
| 9 | 2013-2014 | (Napúa et al., 2016) | Mozambique | Public health facilities | IDI, FGD | Women who tested positive for HIV in ANC services; HCWs | To inform the design of a pilot facility-level intervention to improve patient retention in the context of Option B+. | 5.5 |
| 10 | 2014 | (Zhou, 2016) | Malawi | Public clinics or women's homes | IDI | Women with HIV who started ART in the context of Option B+ | To examine how women understand the prescription for lifelong treatment and what they imagine the benefits to be. | 10 |
| 11 | 2014 | (Buregyeya et al., 2017) | Uganda | Rural health facilities | IDI | Pregnant or lactating women with HIV | To explore experiences of HIV infected pregnant and breastfeeding women regarding barriers and facilitators of uptake and adherence to lifelong ART. | 6.5 |
| 12 | 2014 | (Hanrahan & Williams, 2017) | South Africa | Primary healthcare facilities | SSI | Primary care nurses | To determine RNs’ perspectives on the PMTCT programme as implemented and discover whether improvements could be suggested to guide management in its strategies. | 6.5 |
| 13 | 2014 | (Helova et al., 2017b) | Kenya | 4 health facilities in Kisumu, Migori and Homa Bay counties | IDI, FGD | Pregnant women with HIV, male partners, HCWs | To explore health facility level challenges to Option B+ provision from the perspectives of health care providers and clients at low-resource health facilities. | 8.5 |
| 14 | 2014 | (Leila Katirayi et al., 2017) | Malawi, Zimbabwe | Gov and private health facilities (Malawi); Public health clinics (Zimbabwe) | IDI, FGD | Pregnant and breastfeeding women with HIV and HCWs providing PMTCT services | To identify the lessons learned from implementation of lifelong ART under Option B+ to inform the scaling up of universal ART. | 9.5 |
| 15 | 2014 | (McLean et al., 2017) | Malawi, Tanzania, Uganda | Rural areas: clinic setting or participant’s home | IDI | Pregnant and breastfeeding women with HIV and their male partners | To understand what influences acceptance and adherence to Option B+, and explore whether these influences are specific to pregnancy. | 8 |
| 16 | 2014 | (Rosenberg et al., 2017) | Malawi | ANC clinic within a maternity hospital | IDI | Pregnant women with HIV and their male partners | To address how and why women disclose their HIV status; how the use of a clinic invitation letter to partners was viewed; how men experience disclosure, inviting and tracing processes; and what impact interventions had on couples’ relationships. | 7.5 |
| 17 | 2014 | (Erekaha et al., 2018) | Nigeria | Rural public health clinic catchment areas | SSI | Pregnant women with HIV, HCW, KII | To explore perspectives of PMTCT users and providers on readiness of HIV positive women in rural Nigeria to accept and adhere to lifelong ART under Option B+. | 9 |
| 18 | 2014-2015 | (Chadambuka et al., 2017) | Zimbabwe | Urban & rural health facilities | IDI, FGD, KII | Pregnant and breastfeeding women with HIV, HCWs, community members | To explore the acceptability of Option B+ among pregnant and lactating women to inform health education, promotion activities and client counselling for new guidelines. | 7 |
| 19 | 2014-2015 | (Flax, Hamela, et al., 2017) | Malawi | Urban & rural government clinics | IDI | Women living with HIV - enrolled or LTFU in Option B+ | To identify facilitators and barriers to Option B+ participation and document the duration of breastfeeding among HIV-positive women with children 0-23 months who were participating in, or LTFU from, Option B+. | 7.5 |
| 20 | 2014-2015 | (Gill et al., 2017) | Rwanda | PMTCT program clinics | IDI | Four generations of mothers | To describe attitudes and norms contributing to adherence for women engaged in care and how these might change from around the time of delivery to when their child is at or near the end of breastfeeding. | 8.5 |
| 21 | 2014-2015 | (Kweyamba et al., 2018) | Uganda | Health facilities in rural areas | SSI | Key informants, HCWs | To explore perspectives of health providers on facility preparedness and organization of services in implementation of Option B+ in Central Uganda. | 8 |
| 22 | 2014 - 2015 | (Phiri et al., 2018) | Malawi | Health care facilities | Observation, FGD, SSI | Women >15 initiated on ART in ANC clinics, and subsequently transferred to an ART clinic | To determine motivators for women who have stayed on ART and what barriers they had to overcome to stay on treatment. | 8.5 |
| 23 | 2014-2015 | (Cataldo et al., 2018) | Malawi, Zimbabwe, Uganda | 2 urban and 1 rural health facilities | SSI | Community health workers | To explore tracing strategies deployed in three countries that initiated Option B+, and the consequences of 'tracing' on social relations between CHWs and patients. | 9 |
| 24 | 2015 | (Doherty et al., 2017) | Uganda | Health facilities and a district managers' office; Eastern, Northern and Western regions | KII, FGD | MoH, implementing partners, multilateral agencies, district mgmt, HCW and community members | To explore the impacts of the Option B+ policy shift on the health system. | 7 |
| 25 | 2015 | (Gugsa et al., 2017) | Malawi | District hospital maternity unit | IDI | Pregnant and lactating women initiated on ART under Option B+ | To acquire a deeper understanding of factors affecting women’s treatment-related decision-making during periods of pregnancy and lactation. | 9 |
| 26 | 2015 | (Mulamba et al., 2017) | DRC | Health facilities providing PMTCT services in 6 health zones | SSI | Non-physician care providers who worked in ANCs and pre-school consultations | To understand the perceptions of the providers involved in the implementation of Option B+. | 7.5 |
| 27 | 2015-2016 | (Flax, Yourkavitch, et al., 2017) | Malawi, Uganda | Government health facilities | IDI | Women enrolled in Option B+ or LTFU, Gov HCWs, Stakeholders | To assess gender-related facilitators and barriers to Option B+ participation, in order to inform policy discussions, program implementation and related support for women in PMTCT care. | 9 |
| 28 | 2016 | (Laar et al., 2018) | Ghana | Urban PMTC clinic | IDI | HCW | To explore health care provider views on Option B+ implementation challenges to help inform policy and sustainability on a nationwide scale. | 9 |
| 29 | 2017 | (Bengtson et al., 2020) | Malawi | 2 large urban clinics | FGD, IDI | Women living with HIV, HCW | To explore the reasons for clinic transfers and how transferring affects engagement in care among HIV-infected pregnant women. | 6 |
| 30 | 2017 | (Masereka et al., 2019) | Uganda | 2 rural health centres | FGD | Retained and not retained pregnant and breastfeeding mothers with HIV | To determine the level of retention and factors influencing retention among HIV positive pregnant and breastfeeding mothers. | 6.5 |
| 31 | 2017 | (Sariah et al., 2019) | Tanzania | 3 public HIV care and treatment clinics | IDI | LTFU pregnant and breastfeeding women with HIV | To explore the reasons for LTFU among pregnant and breastfeeding women initiated on Option B+. | 8.5 |
| 32 | 2017-2018 | (Mamba & Hlongwana, 2018) | Swaziland | 2 rural and 1 urban health facilities | IDI | Pregnant women with HIV | To explore reasons that deter pregnant women living with HIV from immediate initiation of ART in the Hhohho Region. | 8 |
